# *catena*-Poly[di-μ_3_-bromido-bis­[(1-ethyl-1*H*-imidazole-κ*N* ^3^)disilver(I)]]

**DOI:** 10.1107/S1600536813016875

**Published:** 2013-06-22

**Authors:** Zhiguo Wang, Qingquan Bian, Ying Guo

**Affiliations:** a Department of Chemistry and Chemical Engineering, Mianyang Normal University, Mianyang 621000, People’s Republic of China

## Abstract

The asymmetric unit of the title coordination complex, [Ag_2_Br_2_(C_5_H_8_N_2_)_2_]_*n*_, comprises a monodentate 1-ethyl­imida­zole ligand, an Ag^+^ cation and a μ_3_-bridging Br^−^ anion, giving a distorted tetra­hedral AgNBr_3_ stereochemistry about the Ag^+^ cation [Ag—N = 2.247 (2) Å and Ag—Br = 2.7372 (4)–2.7523 (4) Å]. Two bridging bromide anions generate the dimeric [Ag_2_Br_2_(C_5_H_8_N)_2_] repeat unit [Ag⋯Ag = 3.0028 (5) Å], while a third Br^−^ anion links the units through corner sharing in an inversion-related Ag_2_Br_2_ association [Ag⋯Ag = 3.0407 (4) Å], generating a one-dimensional ribbon step-polymer structure, extending along the *c* axis.

## Related literature

For general background to *N*-heterocyclic carbenes, see: Arnold (2002 ▶); Lin & Vasam (2004 ▶). For related structures, see: Wang & Lin (1998 ▶); Liu *et al.* (2003 ▶); Helgesson & Jagner (1990 ▶, 1991 ▶); Chen & Liu (2003 ▶).
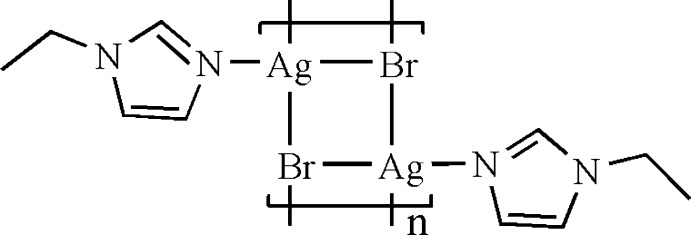


## Experimental

### 

#### Crystal data

[Ag_2_Br_2_(C_5_H_8_N_2_)_2_]
*M* *_r_* = 567.80
Monoclinic, 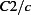

*a* = 15.2489 (15) Å
*b* = 13.9888 (13) Å
*c* = 7.7198 (7) Å
β = 109.809 (1)°
*V* = 1549.3 (3) Å^3^
*Z* = 4
Mo *K*α radiation
μ = 7.67 mm^−1^
*T* = 173 K
0.17 × 0.16 × 0.15 mm

#### Data collection

Bruker APEXII CCD diffractometer
Absorption correction: multi-scan (*SADABS*; Sheldrick, 1996 ▶) *T*_min_ = 0.355, *T*_max_ = 0.392
3840 measured reflections
1362 independent reflections
1315 reflections with *I* > 2σ(*I*)
*R*_int_ = 0.024

#### Refinement

*R*[*F* ^2^ > 2σ(*F* ^2^)] = 0.019
*wR*(*F* ^2^) = 0.048
*S* = 1.05
1362 reflections
83 parameters
H-atom parameters constrained
Δρ_max_ = 0.38 e Å^−3^
Δρ_min_ = −0.68 e Å^−3^

### 

Data collection: *APEX2* (Bruker, 2006 ▶); cell refinement: *SAINT* (Bruker, 2006 ▶); data reduction: *SAINT*; program(s) used to solve structure: *SHELXS97* (Sheldrick, 2008 ▶); program(s) used to refine structure: *SHELXL97* (Sheldrick, 2008 ▶); molecular graphics: *SHELXTL* (Sheldrick, 2008 ▶); software used to prepare material for publication: *SHELXTL*.

## Supplementary Material

Crystal structure: contains datablock(s) I, global. DOI: 10.1107/S1600536813016875/zs2262sup1.cif

Structure factors: contains datablock(s) I. DOI: 10.1107/S1600536813016875/zs2262Isup2.hkl

Additional supplementary materials:  crystallographic information; 3D view; checkCIF report

## Figures and Tables

**Fig. 1.**
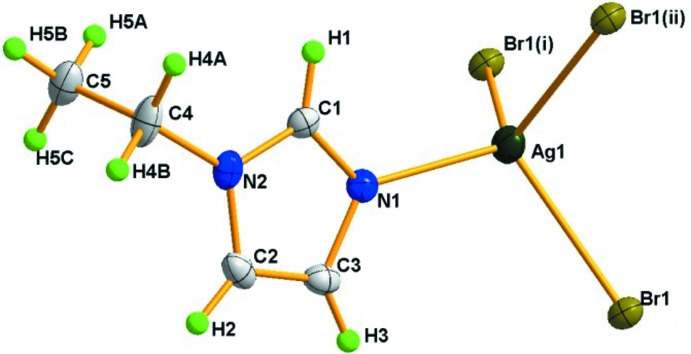
The atom numbering scheme for the contents of the asymmetric unit in the title complex. Displacement ellipsoids are drawn at the 30% probability level. For symmetry codes: (i) -x + 1, y, -z) - 1/2]; (ii) x, -y, z) + 1/2.

**Fig. 2.**
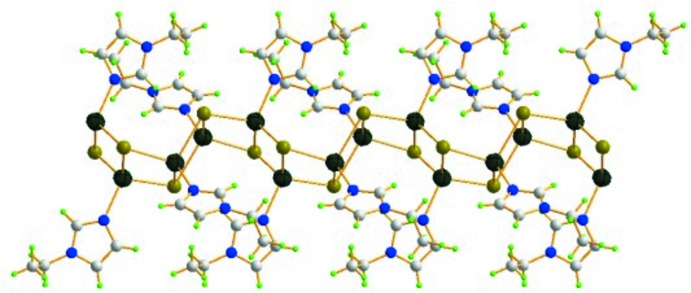
The step-polymeric structure of the title complex, extending along the c axial direction.

## supplementary crystallographic information

### Comment

Silver and other transition metal *N*-heterocyclic carbene complexes have played an important role in development of metal-carbene systems for transmetalation reactions. Recent reviews dealing with silver *N*-heterocyclic carbenes were published by Arnold (2002) and Lin & Vasam (2004). The products differ depending upon reaction conditions and the imidazolium salt used. Deprotonation by use of Ag_2_O has been the most widely used method in the syntheses of *N*-heterocyclic carbene complexes of silver. The procedure can be accomplished using the reaction of Ag_2_O with the imidazolium salt in CH_2_Cl_2_ solution. The 3-diethylbenzole *N*-heterocyclic carbene complexes of silver have been successfully synthesized by the reaction of the 1,3-diethylbenzolium salt with Ag_2_O in CH_2_Cl_2_ (Wang & Lin, 1998). In an attempt to prepare similar *N*-heterocyclic carbene complexes of silver by the reaction of Ag_2_O with 1,2-dibromocyclohexane and 1-ethylimidazole in DMSO solution, we obtained the title compound, [(C_5_H_8_N)_2_Ag_2_Br_2_]_n_, instead and the synthesis and crystal structure are reported herein. Although the stair polymers of [(C_5_H_5_N)_4_Ag_4_I_4_]_n_ (Liu *et al.*, 2003) and 1-allyl-3-methylimidazole carbine silver iodide (Chen & Liu, 2003) have recently been reported, their structural features are different from that of the title complex being formed through triple and quadruple halide bridges with Ag···Ag interactions.

In the title complex the asymmetric unit comprises one monodentate 1-ethylimidazole ligand, an Ag^+^ cation and a doubly bridging Br^-^ anion, giving a distorted tetrahedral AgNBr_3_stereochemistry about silver [Ag—N, 2.247 (2) Å; Ag—Br, 2.7372 (4)–2.7752 (3) Å and bond angle range about Ag of 106.78 (6)–113.55 (5)°] (Fig. 1). These Ag—Br bond distances are considerably longer than those found in the [Ag_2_Br_4_]^2-^ complex anion [2.518 (2) Å] (Helgesson & Jagner, 1990). The Ag1—N1 bond [2.247 (2) Å] is somewhat shorter than 2.335 Å found in the pyridine silver iodide polymer [(C_5_H_5_N)_4_Ag_4_I_4_]_n_ (Liu *et al.*, 2003). The dimeric Ag_2_Br_2_ repeating core unit in the title complex is generated through a double Br bridge, giving an Ag···Ag^i^ separation of 3.0028(r) Å [for symmetry code (i): -*x* + 1, *y*, -*z*) + 1/2]. The four-membered core ring so formed is very similar to that in the complex anion [Ag_4_Br_8_]^4-^(Helgesson & Jagner, 1991).

The basic coomplex is extended into a one-dimensional step-polymer ribbon structure through centrosymmetric Ag—Br and Br—Ag bonds along the *c* axial direction (Fig. 2). Within these cyclic Ag_2_Br_2_ linkages, the Ag···Ag^iii^ separation is 3.0407 (4) Å [for symmetry code (iii): -*x* + 1, -*y* + 1, -*z*].

### Experimental

1,2-Dibromocyclohexane (2.42 g, 10 mmol) was added to a solution of 1-ethylimidazole (1.92 g, 20 mmol) in DMSO (100 ml) at room temperature and stirred for 2 h, after which Ag_2_O (2.32 g, 10 mmol) was added and the mixture was refluxed for 3 h with stirring. The volume of the solution was reduced to 50 ml under vacuum, the residue was removed by filtration and the filtrate was kept at room temperature for a few days. Colorless crystals of the title compound were obtained after slow evaporation (1.74 g, 30% yield). (mp: 335 K). ^1^H NMR(CDCl_3_): 9.42(m,1*H*), 6.88(s, 1*H*, CH), 6.84 (s, 1*H*, CH), 4.54(s, 2*H*, CH_2_), 3.65 (s, 3*H*, CH_3_)p.p.m. Anal. calcd.: C, 21.12; H, 2.82; N, 9.86%; found: C, 21.05; H, 2.76; N, 9.75%.

### Refinement

The H atoms attached to C atoms of the imidazole ring were positioned geometrically and allowed to ride on their parent atoms, with C—H = 0.95 Å and *U*_iso_(H) = 1.2*U*_eq_(C). Methylene and methyl H atoms were likewise positioned geometrically (C—H = 0.99 and 0.98 Å, respectively) and also refined as riding atoms, and *U*_iso_(H) = 1.2*U*_eq_(C)

### Crystal data

**Table:**

[Ag_2_Br_2_(C_5_H_8_N_2_)_2_]	*F*(000) = 1072
*M**_r_* = 567.80	*D*_x_ = 2.434 Mg m^−^^3^
Monoclinic, *C*2/*c*	Melting point: 335 K
Hall symbol: -C 2yc	Mo *K*α radiation, λ = 0.71073 Å
*a* = 15.2489 (15) Å	Cell parameters from 3344 reflections
*b* = 13.9888 (13) Å	θ = 2.8–28.4°
*c* = 7.7198 (7) Å	µ = 7.67 mm^−^^1^
β = 109.809 (1)°	*T* = 173 K
*V* = 1549.3 (3) Å^3^	Block, colourless
*Z* = 4	0.17 × 0.16 × 0.15 mm

### Data collection

**Table:**

Bruker APEXII CCD diffractometer	1362 independent reflections
Radiation source: fine-focus sealed tube	1315 reflections with *I* > 2σ(*I*)
Graphite monochromator	*R*_int_ = 0.024
φ and ω scans	θ_max_ = 25.0°, θ_min_ = 2.9°
Absorption correction: multi-scan (*SADABS*; Sheldrick, 1996)	*h* = −16→18
*T*_min_ = 0.355, *T*_max_ = 0.392	*k* = −16→14
3840 measured reflections	*l* = −6→9

### Refinement

**Table:**

Refinement on *F*^2^	Primary atom site location: structure-invariant direct methods
Least-squares matrix: full	Secondary atom site location: difference Fourier map
*R*[*F*^2^ > 2σ(*F*^2^)] = 0.019	Hydrogen site location: inferred from neighbouring sites
*wR*(*F*^2^) = 0.048	H-atom parameters constrained
*S* = 1.05	*w* = 1/[σ^2^(*F*_o_^2^) + (0.025*P*)^2^ + 1.7996*P*] where *P* = (*F*_o_^2^ + 2*F*_c_^2^)/3
1362 reflections	(Δ/σ)_max_ < 0.001
83 parameters	Δρ_max_ = 0.38 e Å^−^^3^
0 restraints	Δρ_min_ = −0.68 e Å^−^^3^

### Special details

**Table:**

Geometry. All e.s.d.'s (except the e.s.d. in the dihedral angle between two l.s. planes) are estimated using the full covariance matrix. The cell e.s.d.'s are taken into account individually in the estimation of e.s.d.'s in distances, angles and torsion angles; correlations between e.s.d.'s in cell parameters are only used when they are defined by crystal symmetry. An approximate (isotropic) treatment of cell e.s.d.'s is used for estimating e.s.d.'s involving l.s. planes.
Refinement. Refinement of *F*^2^ against ALL reflections. The weighted *R*-factor *wR* and goodness of fit *S* are based on *F*^2^, conventional *R*-factors *R* are based on *F*, with *F* set to zero for negative *F*^2^. The threshold expression of *F*^2^ > σ(*F*^2^) is used only for calculating *R*-factors(gt) *etc*. and is not relevant to the choice of reflections for refinement. *R*-factors based on *F*^2^ are statistically about twice as large as those based on *F*, and *R*- factors based on ALL data will be even larger.

### Fractional atomic coordinates and isotropic or equivalent isotropic displacement parameters (Å^2^)

**Table:**

	*x*	*y*	*z*	*U*_iso_*/*U*_eq_	
Ag1	0.445475 (14)	0.080848 (14)	−0.12049 (3)	0.02369 (10)	
Br1	0.363999 (18)	0.066857 (18)	−0.49464 (4)	0.01975 (10)	
N1	0.39450 (15)	0.21798 (15)	−0.0362 (3)	0.0183 (5)	
N2	0.34342 (14)	0.31681 (15)	0.1304 (3)	0.0204 (5)	
C1	0.38660 (17)	0.23374 (18)	0.1262 (4)	0.0182 (5)	
H1	0.4088	0.1914	0.2280	0.022*	
C2	0.32148 (18)	0.35730 (18)	−0.0411 (4)	0.0236 (6)	
H2	0.2903	0.4163	−0.0808	0.028*	
C3	0.35336 (18)	0.29593 (19)	−0.1426 (4)	0.0216 (6)	
H3	0.3482	0.3052	−0.2676	0.026*	
C4	0.3277 (2)	0.3578 (2)	0.2924 (4)	0.0307 (7)	
H4A	0.3101	0.3061	0.3618	0.037*	
H4B	0.2751	0.4036	0.2514	0.037*	
C5	0.4122 (2)	0.4081 (2)	0.4169 (4)	0.0295 (7)	
H5A	0.4648	0.3635	0.4555	0.044*	
H5B	0.3996	0.4318	0.5256	0.044*	
H5C	0.4275	0.4620	0.3512	0.044*	

### Atomic displacement parameters (Å^2^)

**Table:**

	*U*^11^	*U*^22^	*U*^33^	*U*^12^	*U*^13^	*U*^23^
Ag1	0.02308 (14)	0.02533 (14)	0.02428 (15)	0.00355 (7)	0.01013 (10)	−0.00298 (8)
Br1	0.01675 (15)	0.02566 (16)	0.01700 (17)	0.00117 (9)	0.00595 (12)	0.00022 (10)
N1	0.0171 (11)	0.0193 (10)	0.0203 (12)	−0.0016 (9)	0.0089 (9)	−0.0010 (9)
N2	0.0145 (10)	0.0223 (11)	0.0242 (12)	−0.0017 (9)	0.0064 (9)	−0.0064 (9)
C1	0.0148 (12)	0.0213 (13)	0.0179 (13)	−0.0015 (10)	0.0046 (10)	−0.0001 (10)
C2	0.0193 (13)	0.0171 (13)	0.0312 (15)	−0.0016 (10)	0.0043 (11)	0.0014 (11)
C3	0.0201 (13)	0.0219 (13)	0.0215 (14)	−0.0036 (10)	0.0053 (11)	0.0047 (11)
C4	0.0247 (15)	0.0373 (17)	0.0332 (17)	−0.0033 (12)	0.0138 (13)	−0.0189 (13)
C5	0.0228 (15)	0.0343 (15)	0.0287 (17)	0.0016 (12)	0.0050 (13)	−0.0108 (13)

### Geometric parameters (Å, º)

**Table:**

Ag1—N1	2.247 (2)	N2—C4	1.467 (3)
Ag1—Br1	2.7372 (4)	C1—H1	0.9500
Ag1—Br1^i^	2.7420 (4)	C2—C3	1.358 (4)
Ag1—Br1^ii^	2.7523 (4)	C2—H2	0.9500
Ag1—Ag1^i^	3.0028 (5)	C3—H3	0.9500
Ag1—Ag1^iii^	3.0407 (4)	C4—C5	1.497 (4)
Br1—Ag1^i^	2.7420 (4)	C4—H4A	0.9900
Br1—Ag1^iv^	2.7522 (4)	C4—H4B	0.9900
N1—C1	1.318 (3)	C5—H5A	0.9800
N1—C3	1.381 (3)	C5—H5B	0.9800
N2—C1	1.342 (3)	C5—H5C	0.9800
N2—C2	1.374 (4)		
			
N1—Ag1—Br1	106.78 (6)	C2—N2—C4	127.2 (2)
N1—Ag1—Br1^i^	113.55 (5)	N1—C1—N2	111.8 (2)
Br1—Ag1—Br1^i^	112.899 (9)	N1—C1—H1	124.1
N1—Ag1—Br1^ii^	107.26 (5)	N2—C1—H1	124.1
Br1—Ag1—Br1^ii^	102.771 (10)	C3—C2—N2	106.1 (2)
Br1^i^—Ag1—Br1^ii^	112.797 (10)	C3—C2—H2	126.9
N1—Ag1—Ag1^i^	121.11 (5)	N2—C2—H2	126.9
Br1—Ag1—Ag1^i^	56.845 (11)	C2—C3—N1	109.6 (2)
Br1^i^—Ag1—Ag1^i^	56.690 (10)	C2—C3—H3	125.2
Br1^ii^—Ag1—Ag1^i^	130.781 (8)	N1—C3—H3	125.2
N1—Ag1—Ag1^iii^	128.97 (6)	N2—C4—C5	112.2 (2)
Br1—Ag1—Ag1^iii^	123.435 (12)	N2—C4—H4A	109.2
Br1^i^—Ag1—Ag1^iii^	56.559 (9)	C5—C4—H4A	109.2
Br1^ii^—Ag1—Ag1^iii^	56.238 (11)	N2—C4—H4B	109.2
Ag1^i^—Ag1—Ag1^iii^	95.508 (11)	C5—C4—H4B	109.2
Ag1—Br1—Ag1^i^	66.464 (9)	H4A—C4—H4B	107.9
Ag1—Br1—Ag1^iv^	109.172 (11)	C4—C5—H5A	109.5
Ag1^i^—Br1—Ag1^iv^	67.204 (10)	C4—C5—H5B	109.5
C1—N1—C3	105.3 (2)	H5A—C5—H5B	109.5
C1—N1—Ag1	124.76 (17)	C4—C5—H5C	109.5
C3—N1—Ag1	129.32 (18)	H5A—C5—H5C	109.5
C1—N2—C2	107.1 (2)	H5B—C5—H5C	109.5
C1—N2—C4	125.6 (2)		
			
N1—Ag1—Br1—Ag1^i^	116.60 (6)	Br1^i^—Ag1—N1—C3	107.5 (2)
Br1^i^—Ag1—Br1—Ag1^i^	−8.881 (15)	Br1^ii^—Ag1—N1—C3	−127.1 (2)
Br1^ii^—Ag1—Br1—Ag1^i^	−130.686 (9)	Ag1^i^—Ag1—N1—C3	43.4 (2)
Ag1^iii^—Ag1—Br1—Ag1^i^	−72.907 (15)	Ag1^iii^—Ag1—N1—C3	172.66 (18)
N1—Ag1—Br1—Ag1^iv^	169.81 (6)	C3—N1—C1—N2	−0.2 (3)
Br1^i^—Ag1—Br1—Ag1^iv^	44.330 (16)	Ag1—N1—C1—N2	−172.05 (16)
Br1^ii^—Ag1—Br1—Ag1^iv^	−77.474 (18)	C2—N2—C1—N1	0.3 (3)
Ag1^i^—Ag1—Br1—Ag1^iv^	53.212 (9)	C4—N2—C1—N1	−176.8 (2)
Ag1^iii^—Ag1—Br1—Ag1^iv^	−19.696 (19)	C1—N2—C2—C3	−0.3 (3)
Br1—Ag1—N1—C1	152.24 (18)	C4—N2—C2—C3	176.8 (2)
Br1^i^—Ag1—N1—C1	−82.7 (2)	N2—C2—C3—N1	0.1 (3)
Br1^ii^—Ag1—N1—C1	42.6 (2)	C1—N1—C3—C2	0.0 (3)
Ag1^i^—Ag1—N1—C1	−146.79 (17)	Ag1—N1—C3—C2	171.36 (17)
Ag1^iii^—Ag1—N1—C1	−17.6 (2)	C1—N2—C4—C5	80.9 (3)
Br1—Ag1—N1—C3	−17.5 (2)	C2—N2—C4—C5	−95.7 (3)

Symmetry codes: (i) −*x*+1, *y*, −*z*−1/2; (ii) *x*, −*y*, *z*+1/2; (iii) −*x*+1, −*y*, −*z*; (iv) *x*, −*y*, *z*−1/2.
